# Supplementary material for: A population-based study of administrative data linkage to measure melanoma surgical and pathology quality
Source: PLoS One. 2022 Feb 18;17(2):e0263713. doi: 10.1371/journal.pone.0263713 (PMC8856577; doi:10.1371/journal.pone.0263713)
Supplement: S1 Appendix — (DOCX) [file pone.0263713.s001.docx]

**Appendix 1.** Identification of invasive cutaneous melanoma patients and their pathology records in Ontario from January 1, 2007 to December 31, 2012.

Pathology database with a 65% random sample of invasive cutaneous melanoma cases diagnosed in January 2007-December 2012 in Ontario

**N=9,212 patients (9,802 cases)**

Linkage with ICES Ontario Cancer Registry (OCR)

**N=8,877 patients with 9,462 cases**

Non-valid IKNs in ICES OCR database

**N=259 patients (261 cases)**

Lapses in OHIP coverage in

5-year lookback from diagnostic date

**N=289 patients**

Mismatches in age and sex, 19 years or younger, and out-of-province residents

**N=102 patients**

Quality indicators for first primary diagnosis

**N=8,045 patients**

Missing pathology records at first diagnosis

**N=438 patients**

In situ melanoma cases at first diagnosis

**N=386 patients**

Melanoma pathology platform

**N=7,654 patients**

Non-applicable or unreliable pathology records

**N=76 patients (79 cases)**

Core/FNA only biopsies at first diagnosis

**N=8 patients**
