# Supplementary material for: A population-based study of administrative data linkage to measure melanoma surgical and pathology quality
Source: PLoS One. 2022 Feb 18;17(2):e0263713. doi: 10.1371/journal.pone.0263713 (PMC8856577; doi:10.1371/journal.pone.0263713)
Supplement: S2 Appendix — (DOCX) [file pone.0263713.s002.docx]

**Appendix 2**. Ontario Health Insurance Plan (OHIP) billing codes.

| **Procedure Description** | **Billing Codes** |
| --- | --- |
| Previous lymph node surgical experience for specified surgeons | E505, E546, R105, R910-R915, S043-S045, S776, Z405, Z406, Z411, Z578, R010 |
| Abdomino-Pelvic CT | X409, X410, X126, X231-X234 |
| Lymphoscintigraphy | J861, J661, J866, J010 |
