# Supplementary material for: A population-based study of administrative data linkage to measure melanoma surgical and pathology quality
Source: PLoS One. 2022 Feb 18;17(2):e0263713. doi: 10.1371/journal.pone.0263713 (PMC8856577; doi:10.1371/journal.pone.0263713)
Supplement: Attachment — Submitted filename: Reviewer Response.docx [file pone.0263713.s003.docx]

**Response to Reviewers**

A Population-Based Study of Administrative Data Linkage to Measure Melanoma Surgical and Pathology Quality

McKay DR, Nguyen P, Wang A, Hanna TP

Running title: Melanoma Quality Indicators

**Reviewer #1: The Authors assessed the quality of melanoma surgical management and pathologic reporting in a retrospective population-based cohort of 7,654 melanoma cases observed in the years 2007-2012. They identified variation in surgery and suboptimal pathology reporting according to quality indicators relevant to practice at that time. Consultations with surgeons offering SLNB occurred in only two thirds of eligible cases, and far less received SLNB. Nearly one quarter of patients had less than the optimal number of nodes in report in non-SLNB dissection. Although the topic is potentially interesting, the fact that the study dates back to the years 2007-2012 represents a major weakness. The obtained results lack of interest since they do not reflect the current scenario of melanoma management worldwide.**

We have re-framed the study to better highlight the relevant and distinctive elements of it, by emphasizing that the work is fundamentally a pilot study of using a linked population-based administrative data set to explore the feasibility of measuring melanoma quality of care with administrative data. We have expanded the content where we explore the feasibility of each specific consensus-based quality indicator, as this is an important part of exploring the feasibility of using administrative data for continuous quality measurement (e.g. see the new Table 2). We have accordingly edited the discussion and conclusions to reflect this.

We have also focused discussion of the quality indicator measurements on issues that could reflect a quality issue still relevant today. We spend time in the discussion of the paper reviewing how a comprehensive suite of quality indicators could be explored with current strategies moving forward (e.g. sampling, natural language processing).

Reviewer #2: Introduction
**“Notably, it is a disease managed by many different physician specialists”. This statement should be better discussed. Why this is a cause of treatment inequity? Have you other explanations for this?**

We have now highlighted that variety of physicians required to treat melanoma is not fundamentally negative, but does pose challenges to ensuring optimal quality of care:

‘Notably, it is a disease managed by many different physician specialists. This speaks to the diversity of expertise required to optimally treat melanoma, and also the challenge in optimizing the quality of care.’

**The authors should better introduce the issue of quality. Please, briefly summarize the potential causes of melanoma treatment inequity and the lack of standardization. Moreover, the authors should underline why quality assurance is important in melanoma care. I suggest reading and cite this interesting review “Pasquali S. et al.Measuring the quality of melanoma surgery - Highlighting issues with standardization and quality assurance of care in surgical oncology. Eur J Surg Oncol. 2017 Mar;43(3):561-571. doi: 10.1016/j.ejso.2016.06.397. Epub 2016 Jun 29. PMID: 27422583”**

Thank-you for the very constructive feedback. We have completely re-written the introduction to address these points. We have referenced Pasquali et al, an excellent source. The introduction now reads:

‘Introduction

Concerns regarding surgical treatment quality for melanoma have been raised in Canada and other jurisdictions [1-3]. A population-based study in the Canadian province of Ontario suggests significant variations exist in melanoma surgical management, potentially leading to inadequate care for some, with greater likelihood of inadequate treatment with advanced age, female sex, and in certain jurisdictions [4]. At the same time, stage and outcome have been reported to vary by patient factors such as age, sex and area-level socioeconomic status, suggesting a need to optimize the quality of care between groups to ensure optimal outcomes [2, 5].

Surgery and pathology quality improvement is essential. High quality surgery and pathology reporting are necessary to guide stage-based treatment decisions for optimal melanoma care, including choice of adjuvant systemic therapy that can improve cancer outcomes, including survival [6-9]. Improving the quality of care can also reduce waste, and improve the cost-effectiveness of care for melanoma [10]. Notably, formally developed quality indictors validated through expert consensus have been developed to assess melanoma care quality, though they have not been explored as broadly as for other cancers [11].

Continuously measuring and reporting on quality indicators for melanoma and other cancers is crucial, as it may contribute to reducing inappropriate practice variation, and improving outcomes [12]. However, collecting and compiling the information required to do so can be resource intensive, time consuming and costly. We thus set out to explore the utility and feasibility of linked routinely collected health data to capture key elements of quality of surgical management and pathologic reporting in a single payer, universal health setting. To do so, we set out to describe melanoma quality of care in Ontario, using a well-characterized population-based sample with detailed pathology report information [3]. Linked administrative data could provide a means to efficiently and continuously measure variation in quality of care, to improve the quality of care for those diagnosed with melanoma.’

**Although implicit, the aim of the study is not clear and should be better defined.**

We have altered the title to be more explicit in the aim of our study. We have also edited the background/rationale to better achieve this.

**Methods
Please replace this redundant explanation “selected to provide a representative picture of the diagnosis and surgical treatment of melanoma across the province of Ontario during the study period”. Please limit this section on the description of method used.**

Thank-you, this sentence has been deleted.

**The selection process is partly difficult to follow and not completely clear. Please list the inclusion and exclusion criteria more clearly.**

To improve the clarity of the process, the methods were edited and expanded. To provide transparency, a new paragraph was added to the results, summarizing reasons that quality indicators were deemed feasible (or not) for measurement with administrative data. These all related to various forms of missing data (clinical, pathologic or structural). The quality indicators, reasons for feasibility or lack of feasibility, and type of missing data (if present) was catalogued in a new exhaustive table (Table 2).

**“Available pathology reports for all patients were provided from CCO and abstracted according to a standardized algorithm”, which algorithm?**

We have now specified that the algorithm was developed by our research group specifically for the provincial melanoma pathology database:

‘Available pathology reports for all patients were provided from CCO and abstracted according to a standardized algorithm developed at the Queen’s University Division of Cancer Care and Epidemiology, and deterministically linked to each patient’s OCR record according to their group ID, a unique identifier.’

**In general, in this section, the authors take many things as granted. The authors should better explain why these indicators were selected for the study.**

The selection process for quality indicators has been clarified in the methods. We emphasize that the metrics were chosen to provide a range of valid and contemporaneous quality indicators on which to assess the feasibility of measurement with administrative data.

**Why recognized quality indicators as False negative rate after SLNB or Positivity rate after SLNB were not considered? Are they difficult to be retrieved from the data base?**

For this pilot study exploring feasibility, we used two sources providing a panel of consensus-based melanoma quality indicators. False negative and SLNB positivity were not identified within these sources, and so were not included. In practice, as these require data from multiple sources, and for the false negative rate, extended follow-up, these are more complex to measure, and this may have been why consensus panels did not identify these. We agree these are important quality metrics, and so have included a comment on this point in the discussion under the limitations section:

There are limitations to our study. Some metrics that are relevant to the clinical community may not have been included in the recommended consensus-based quality indicators we considered. For example, the false negative rate after SLNB or SLNB positivity rate. We note that the former requires multiple data sources and extended follow-up, though is technically feasible with appropriate data linkage.

**Results
“invasive patients” What do you mean? Patients with invasive melanoma? Please modify.**

We have modified this for clarity: We identified a cohort of 7,654 adult patients with invasive melanoma, provincial health coverage and linked pathology records from 8,877 linkable patients with 9,462 pathology records (Appendix 1, Table 1).  **Discussion
In this section the topic of inequality in melanoma management is well discussed. The results are interesting and limitations clearly underlined.**

Thank-you.

**Regarding number of lymph nodes after dissection, I suggest to cite and discuss the following study “Rossi CR, Number of excised lymph nodes as a quality assurance measure for lymphadenectomy in melanoma. JAMA Surg. 2014 Jul;149(7):700-6. doi: 10.1001/jamasurg.2013.5676. PMID: 24804856.. The number of lymph nodes retrieved are not only an indicator of good practice but also may have an impact on prognosis.**

Thank-you we have cited this study and provided some discussion. We now state:

‘There are limitations to our study. Some metrics that are relevant to the clinical community may not have been included in the recommended consensus-based quality indicators we considered. For example, the false negative rate after SLNB or SLNB positivity rate. We note that the former requires multiple data sources and extended follow-up, though is technically feasible with appropriate data linkage. We also note that recommendations on the optimal number of lymph nodes to be removed varies to some degree between studies, though the purpose of our present investigation was to explore feasibility and utility of using data linkage for quality measurement, rather than to determine the best metric [29]. We also note that the impact of lymph node dissection surgery and pathology reporting on outcomes requires further investigation.’

**Do you have any idea how many patients are treated outside the registry? This may be a further limitation of the study.**

The 65% random sample is population-based, and is within the context of a universal health care system. Thus the practice outside of the study cohort should be analogous.

We have now emphasized this in the methods:

‘This pilot study utilized a previously developed retrospective population-based cohort. A 65% random sample of all invasive melanoma cases diagnosed in Ontario between January 1, 2007 and December 31, 2012 that were recorded in the Ontario Cancer Registry (OCR) was used for this study. Completely abstracted population-based pathology data for later years was not available. The OCR is administered by Cancer Care Ontario (CCO), the provincial cancer agency associated with Ontario’s single payer universal health system. The random sample thus provides insights into the management of the complete population of people diagnosed with melanoma over the study period.’

**Moreover, the authors should discuss how to manage the results of the study. It is well known that simply publishing and disseminating guidelines are not sufficient to ensure homogeneity and quality of surgical treatment. Promoting a program for quality control of pathology and surgery in melanoma following these quality indicators should be the next step. Which initiatives have the authors in mind in the next near future? Please better underline the future perspectives of the study.**

Next steps are now discussed in the methods:

‘To support a program of quality improvement for melanoma pathology and surgery, continuous measurement and reporting of a comprehensive suite of melanoma quality indictors is required. Though the available quality metrics showed important gaps in quality of care requiring attention, we importantly found that only a minority of quality metrics could be measured with meticulously collected linked administrative data sources. How can this be addressed? With the rise of electronic synoptic reporting, some information sources are being more efficiently and completely collected in our jurisdiction and many others. However, other important variables require attention. For example, clinical margin width is only collected systematically in the individual patient chart. Manual abstraction for all patients is time- and resource-intensive. Other alternatives are routine sampling of a small, representative subset of patients from each institution on an ongoing basis. This approach has been used in oncology, though still requires a substantial amount of time and coordination. Artificial intelligence (AI) with natural language processing may provide a solution for variables that are routinely collected such as margin width. This requires a comprehensive data infrastructure and human capacity to ensure the training of algorithms to ensure complete and accurate data capture. We note that in our jurisdiction, efforts are underway to harness AI approaches to capture non-melanoma skin cancer, and these approaches could be transferred to melanoma populations.’

**References
I suggest to include the following studies;
Sommariva et al.Eur J Surg Oncol. 2018 Nov;44(11):1773-1778. doi: 10.1016/j.ejso.2018.06.020. Epub 2018 Jul 3. PMID: 30001892.
Sommariva et al. Standardization and quality control of surgical treatment of cutaneous melanoma: looking for consensus of the Italian melanoma intergroup. Eur J Surg Oncol. 2015 Jan;41(1):148-56. doi: 10.1016/j.ejso.2014.07.035. Epub 2014 Jul 26. PMID: 25108812.**

Thank-you. We have now included this excellent study in our references. We note results of this study when discussing the limitations of our study, in particular the included quality indicators, and the variation in recommended quality indicators between studies. We emphasize that the purpose of our study was to investigate the feasibility and utility of using linked administrative data sources, rather than to determine the optimal quality metric and benchmark value for these.
