# Supplementary material for: A population-based study of administrative data linkage to measure melanoma surgical and pathology quality
Source: PLoS One. 2022 Feb 18;17(2):e0263713. doi: 10.1371/journal.pone.0263713 (PMC8856577; doi:10.1371/journal.pone.0263713)
Supplement: Attachment — Submitted filename: Response to Reviewers.docx [file pone.0263713.s004.docx]

**PONE-D-21-29441**

**A Population-Based Study of Administrative Data Linkage to Measure Melanoma Surgical and Pathology Quality**

**PLOS ONE**

**1. Please ensure that your manuscript meets PLOS ONE's style requirements, including those for file naming. The PLOS ONE style templates can be found at**

**https://journals.plos.org/plosone/s/file?id=wjVg/PLOSOne_formatting_sample_main_body.pdf and**

**https://journals.plos.org/plosone/s/file?id=ba62/PLOSOne_formatting_sample_title_authors_affiliations.pdf**

The manuscript has been revised in according with PLOS ONE’s style requirements.

**2. Thank you for stating the following in the Acknowledgments Section of your manuscript:**

**"This study was supported by ICES, which is funded by an annual grant from the Ontario Ministry of Health and Long-Term Care (MOHLTC). The opinions, results and conclusions reported in this paper are those of the authors and are independent from the funding sources. No endorsement by ICES or the Ontario MOHLTC is intended or should be inferred. Parts of this material are based on data and/or information compiled and provided by CIHI. However, the analyses, conclusions, opinions and statements expressed in the material are those of the author(s), and not necessarily those of CIHI. Parts of this material are based on data and information provided by Cancer Care Ontario (CCO). The opinions, results, view, and conclusions reported in this paper are those of the authors and do not necessarily reflect those of CCO. No endorsement by CCO is intended or should be inferred. These data were linked using unique encoded identifiers and analyzed at ICES.**

**T.P.H. holds a research chair provided by the Ontario Institute for Cancer Research through funding provided by the Government of Ontario (#IA-035)"**

**We note that you have provided funding information that is not currently declared in your Funding Statement. However, funding information should not appear in the Acknowledgments section or other areas of your manuscript. We will only publish funding information present in the Funding Statement section of the online submission form.**

Thank-you. We have removed the statement about funding information and added it to our Funding Statement (see below).

**Please remove any funding-related text from the manuscript and let us know how you would like to update your Funding Statement.**

This has been removed. Please list our statement as:

This study was supported by Kingston Health Sciences Centre Foundation (Fund 70174). Awarded to DM, TH, AW. www.uhkf.ca. The sponsor played no role in the study design, data collection and analysis, decision to publish, or preparation of the manuscript. This study was also supported by ICES, which is funded by an annual grant from the Ontario Ministry of Health and Long-Term Care (MOHLTC). The opinions, results and conclusions reported in this paper are those of the authors and are independent from the funding sources. No endorsement by ICES or the Ontario MOHLTC is intended or should be inferred. T.P.H. holds a research chair provided by the Ontario Institute for Cancer Research through funding provided by the Government of Ontario (#IA-035).

**The Authors did a great job in improving their manuscript after previous submission. Here are somme comments that are meant to highlight findings in the discussion section.**

Thank-you

**Given their expertise, can Authors provide in the discussion information about implications for clinical practice and research of their findings?**

We have added a discussion on potential trends in use of SLNB given lower SLNB rates among some groups:

Nevertheless, we hypothesize that SLNB referral has increased due to availability of new effective adjuvant targeted and immune-based therapies where pathologically confirmed lymph node involvement is required for funding. The additional impact of MSLT-II and the German Dermatologic Cooperative Oncology Group (DeCOG) trial SLNB trial on SLNB rates are less clear. These studies suggest that completion dissection increases regional disease control and provide prognostic information for SLNB positive patients, without increasing melanoma-specific survival (MSLT-II) or distant metastasis-free survival (DeCOG) [27, 28]. Whether this evidence supporting SLNB alone without completion dissection for SLNB positive patients would increase use of SLNB by possibly removing hesitance to use SLNB is unclear. This requires further confirmatory study.

We have also added a discussion on the role of adequate completion lymph node dissection given the low performance on these quality indicators in our study:

Furthermore, the number lymph nodes dissected for non-SLNB procedures failed to meet established quality metrics for between 18% (inguinal basin) to 33% (cervical basin) of cases. The question of adequacy of resection is not novel to this study [21-24]. Spillane et al in a retrospective review of the number of nodes taken at the Sydney Melanoma Unit (SMU) demonstrated that over 90% of cases had greater or equal to 7 and 10 nodes in the specimen for the groin and axilla respectively. For the neck they subdivided results on the basis of levels taken: for those cases where 3 or less levels were excised greater than 90% of patients had 6 or more nodes taken and for neck dissections of four or more levels greater than 90% of patients has 20 or more nodes taken [22]. There were similar findings in Rossi et al’s large patient series from nine Italian Melanoma Intergroup institutions [29]. If we consider the SMU as a center of expertise and directly compare provincial data on the number of nodes resected, outcomes fall below the proposed 90% threshold number of nodes taken for all regional basins. We note that others have found similar under-performance for LND/CLND benchmarks in large population data. For example Bilimoria et al found achievement of the LND/CLND consensus-based benchmarks were low in the United States National Cancer Database [11]. Adequacy of surgical management of these basins bears further scrutiny for these populations.

There is no prospective data for melanoma linking these numbers to recurrence-free survival, but the numbers themselves can be used as a proxy for thoroughness of treatment and pathologic evaluation. Regular evaluation of lymph node dissection quality remains important in the MSLT-II era. This study showed no melanoma-specific-survival advantage for patients who went on to regional node resection following positive sentinel node biopsy compared to nodal observation with ultrasonography [27]. Importantly, SLNB positive patients now managed with ultrasound follow-up rather than CLND will commonly develop lymph node recurrence (26% by five years in MSLT-II), and for these patients, the quality of salvage lymph node dissection bears importance. Measurement of appropriate imaging follow-up for SLNB positive patients without CLND will also be an important quality indicator going forward.

**Among missing information, histopathologic margins are very important. Can you please comment on this missing information?**

We have now added to the discussion:

‘Six of the indicators not measurable with administrative data related to margins as we did not have access to surgical reports documenting the clinical excision margin (5/6 metrics), or pathology report information for excision samples with no residual melanoma that would have confirmed negative margins in many cases (1/6 metrics).’

**How these results compared with existing literature?**

We have added a comparison to selected indicators from the Sydney Melanoma Unit and commented on findings from Rossi et al and Bilimoria et al:

Furthermore, the number lymph nodes dissected for non-SLNB procedures failed to meet established quality metrics for between 18% (inguinal basin) to 33% (cervical basin) of cases. The question of adequacy of resection is not novel to this study [21-24]. Spillane et al in a retrospective review of the number of nodes taken at the Sydney Melanoma Unit (SMU) demonstrated that over 90% of cases had greater or equal to 7 and 10 nodes in the specimen for the groin and axilla respectively. For the neck they subdivided results on the basis of levels taken: for those cases where 3 or less levels were excised greater than 90% of patients had 6 or more nodes taken and for neck dissections of four or more levels greater than 90% of patients has 20 or more nodes taken [22]. There were similar findings in Rossi et al’s large patient series from nine Italian Melanoma Intergroup institutions [27]. If we consider the SMU as a center of expertise and directly compare provincial data on the number of nodes resected, outcomes fall below the proposed 90% threshold number of nodes taken for all regional basins. We note that others have found similar under-performance for LND/CLND benchmarks in large population data. For example Bilimoria et al found achievement of the LND/CLND consensus-based benchmarks were low in the United States National Cancer Database [11]. Adequacy of surgical management of these basins bears further scrutiny for these populations.

**How Authors expect their results to be in a more contemporary patient series? For instance, do they expect rate of SLNB performance even lower after results of the German trial and MSLT-II? Which are the implications for adjuvant therapies? it would be interesting to have Authors view.**

We have edited the manuscript to discuss these interesting questions:

For other metrics, it is not as certain that improvements have been made since the time of our pilot cohort. For example, SLNB and the beneficial role of lymphoscintigraphy for melanoma have been well documented in the literature for almost 20 years [20-23]. The final analysis of the MLST-1 trial only became available in 2014 confirming the benefits of SLNB; however, reports on the benefits of SLNB in this trial span the era under study [24-26]. By considering whether the surgeon seen in consultation had experience performing SLNB based on previous billing code use, we saw that only 2,726 (66.0%) of Stage IB-II and T1b-T4a patients eligible for a sentinel node biopsy were seen by such a surgeon, and less for high-risk <1mm patients (647). *Nevertheless, we hypothesize that SLNB referral has increased due to availability of new effective adjuvant targeted and immune-based therapies where pathologically confirmed lymph node involvement is required for funding. The additional impact of MLST-II and the German SLNB trial on SLNB rates are less clear. These studies suggest that completion dissection increases regional disease control and provide prognostic information for SLNB positive patients, without increasing melanoma-specific survival. Whether this evidence supporting SLNB alone without completion dissection for SLNB positive patients would increase use of SLNB by possibly removing hesitance to use SLNB is unclear. This requires further confirmatory study.*

**The main comment is about the final conclusion of the manuscript. There are differences between abstract and full-text which should be report consistent information. I would rather reccoment to use the conclusion of the full-text manuscript which look more informative to me.**

We have revised the abstract conclusion to align it with the full-text conclusion:

**Conclusions:** We found that use of linked administrative data sources is feasible for measurement of melanoma quality in some cases. In those cases, findings suggest opportunities for quality improvement. Consultation with surgeons offering SLNB was limited, and pathology report completeness was sub-optimal, but was prior to routine synoptic reporting. However, to measure more quality indicators, text-based data sources will require alternative approaches to manual collection such as natural language processing or standardized collection. We recommend development of robust data platforms to support continuous re-evaluation of melanoma quality indicators, with the goal of optimizing quality of care for melanoma patients on an ongoing basis.

**Reviewer #1: The authors have performed substantial changes in the text, especially in the introduction and discussione section, as required by my previous review. The limits of the study are clearly stated and the implications for further improvements in the topic of quality of care in melanoma provided.**

**No further changes are needed**

Thank-you. We appreciate Reviewer #1’s time and comments.

.
